# N-Acetylcysteine Interference With Creatinine Measurement: An In Vitro Analysis

**DOI:** 10.1016/j.ekir.2021.04.006

**Published:** 2021-04-19

**Authors:** Christopher McCudden, Edward G. Clark, Ayub Akbari, Jennifer Kong, Salmaan Kanji, Swapnil Hiremath

**Affiliations:** 1 Department of Pathology and Laboratory Medicine, University of Ottawa, Ottawa, Ontario, Canada; 2 Division of Biochemistry, The Ottawa Hospital, Ottawa, Ontario, Canada; 3 Department of Medicine, University of Ottawa, Ottawa, Ontario, Canada; 4 Division of Nephrology, Ottawa Hospital Research Institute, Ontario, Canada; 5 Department of Pharmacy, The Ottawa Hospital, Ottawa, Ontario, Canada; 6 Ottawa Hospital Research Institute, Ottawa, Ontario, Canada

N-acetylcysteine (NAC) is a derivative of the cysteine amino acid, and was introduced in the 1960s primarily as a mucolytic,^1^ for which it is still used orally as well as in a nebulized form. It also can serve as a substrate for glutathione synthesis, which has an antioxidant property, and is depleted in states such as acetaminophen (or paracetamol) intoxication.^2^ Hence, NAC is used as an antidote in the intravenous form in this setting, as well as increasingly also in severe alcoholic hepatitis, with some evidence of benefit.^3^ In addition, it became popular to prevent contrast-induced acute kidney injury (CI-AKI) until it was finally shown to be not beneficial in a large definitive trial.^4^ However, the data on NAC and prevention of AKI in this and other settings, such as perioperative AKI, is decidedly mixed and a clear explanation for this heterogeneity has not yet been conclusively reported.

One possible explanation for the discrepancy in outcomes with NAC in the AKI literature could be related to the measurement of creatinine, since notably in the CI-AKI trials, the benefit was mostly seen in trials with a change in creatinine as an outcome, and not in clinical outcomes such as need for dialysis or death. Serum creatinine can be measured in different ways. The 2 commonly used methods to measure creatinine level are the modified Jaffe colorimetric assay and the enzymatic method.^5^^,^^6^ The enzymatic method is regarded as more of an accurate method for staging chronic kidney disease and less susceptible to interference when compared with the Jaffe method.^7^ However, uncertainty remains because both methods have not been performed in the same subjects, and most studies are in healthy humans, and any effect of NAC at higher level of creatinine or in patients with chronic kidney disease is not clearly known.

With the hypothesis that the method of measuring creatinine may influence the interference of NAC on creatinine measurement, we conducted this *in vitro* study by adding specific concentration of NAC to blood samples with known different levels of creatinine and measuring creatinine again with different methods, as well as measuring other markers of kidney function.

## Results

A total of 24 samples of waste blood plasma were used for analysis. Creatinine pools were divided into 3 levels: low, 50 μmol/l (*n* = 8); medium, 100 μmol/l (*n* = 8); high, 200 μmol/l (*n* = 8) (Table 1). Similar pools were created for cystatin-C (1, 2, 5 mg/l concentrations), and beta-trace protein (1, 2, 5 mg/l concentrations). Specific amount of NAC was then added to each sample to achieve low to high concentrations of NAC, ranging from 0 to 2000 μg/ml (see Table 1 for details).

**Table 1:** Detailed sample preparation procedure

Sample ID	Creatinine Pool	EDTA Buffer Add (μl)	Creatinine Pool (μl)	2 mg/ml Stock NAC Add (μl)	20 mg/ml Stock NAC Add (μl)	NAC Final Concentration (μg/ml)	Creatinine Expected (μmol/l)
1	Low	100	900	0		0	50
2	Low	87.5	900	12.5		25	50
3	Low	75	900	25		50	50
4	Low	50	900	50		100	50
5	Low	90	900		10	200	50
6	Low	75	900		25	500	50
7	Low	50	900		50	1000	50
8	Low	0	900		100	2000	50
9	Medium	100	900	0		0	100
10	Medium	87.5	900	12.5		25	100
11	Medium	75	900	25		50	100
12	Medium	50	900	50		100	100
13	Medium	90	900		10	200	100
14	Medium	75	900		25	500	100
15	Medium	50	900		50	1000	100
16	Medium	0	900		100	2000	100
17	High	100	900	0		0	200
18	High	87.5	900	12.5		25	200
19	High	75	900	25		50	200
20	High	50	900	50		100	200
21	High	90	900		10	200	200
22	High	75	900		25	500	200
23	High	50	900		50	1000	200
24	High	0	900		100	2000	200

NAC, N-acetylcysteine.

Add 900 μl of creatinine pool for total volume of 1000 μl for each sample

**Figure 1:**
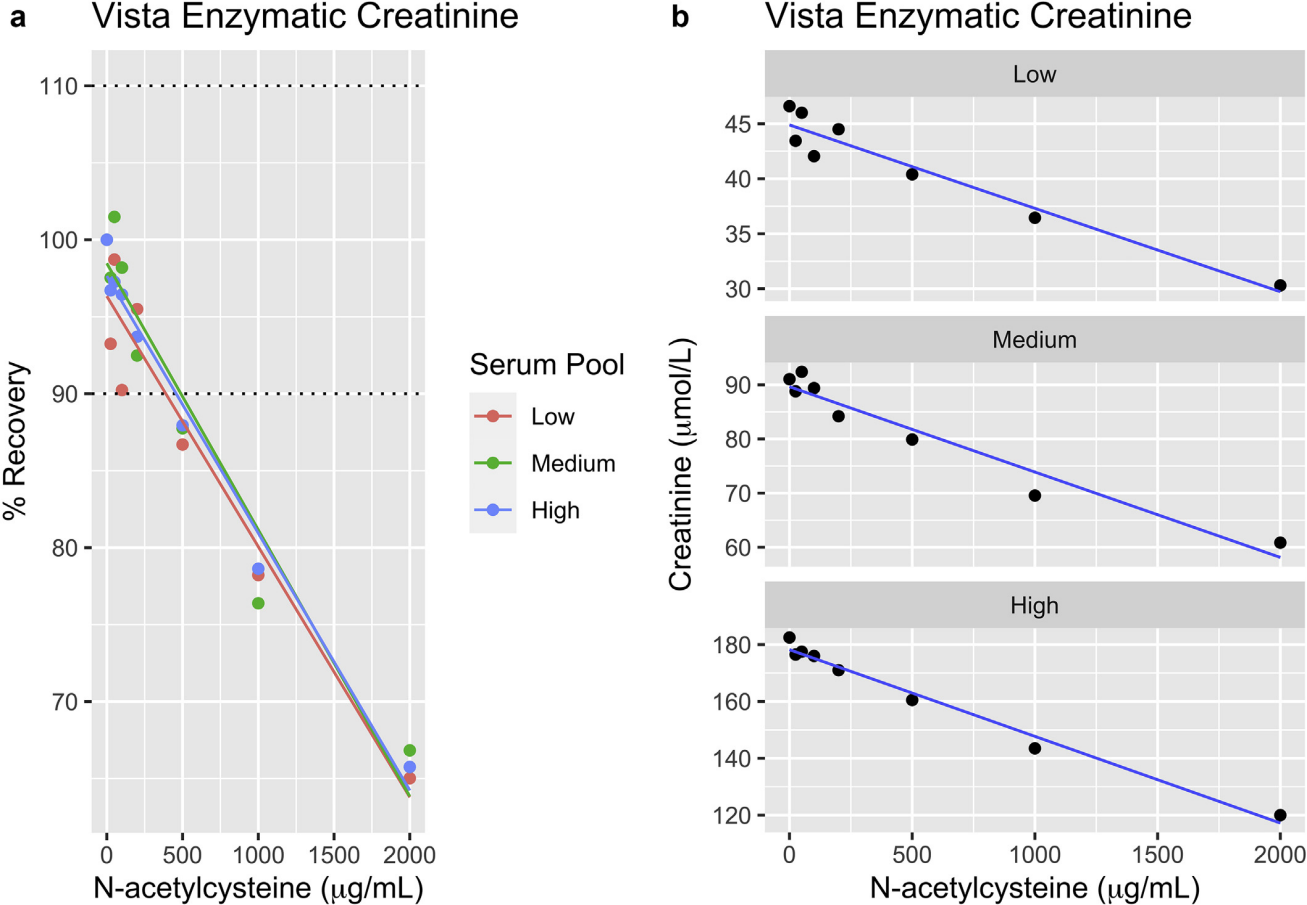
Effect of N-acetylcysteine (NAC) on enzymatic creatinine. (a) Effect of N-acetylcysteine on the absolute concentration of the Siemens enzymatic creatinine. (b). Recovery of Siemens enzymatic with NAC.

Addition of NAC had a dose-dependent effect on creatinine as measured by the enzymatic assay, with a decrease in measured creatinine at the highest NAC concentration ranging from –15 μmol/l in the low creatinine pool (i.e., creatinine being measured at 35 instead of 50 μmol/l) and 60 μmol/l (from 200 to 140 μmol/l) for the high creatinine pool. The negative bias was greater than 10% at NAC concentration of ≥ 400 μg/ml (see interferogram Figure 1a and b). This effect was consistent across all creatinine concentrations tested (50, 100, 200 μmol/l). Unlike the enzymatic assay, the Jaffe creatinine method was unaffected by NAC addition (Supplementary Figure S1).

Similarly, there was no effect of NAC addition at any concentration of NAC for measurement of beta-trace protein (Supplementary Figure S2) or cystatin-C (Supplementary Figure S3).

## Discussion

In this *in vitro* study, we demonstrated that addition of NAC interferes with, and lowers, plasma creatinine as measured only by the enzymatic assay, and not the Jaffe method. There is no interference with cystatin-C or beta-trace protein. This interference is dose-dependent, and is greater than 10% at NAC concentrations >400 μg/l. The interference was not dependent on the baseline serum creatinine level of the sample, and was similar at low or high levels.

The results are also significant because of ongoing use of NAC in other settings and in clinical research. Clinical registries still report ongoing trials on NAC with kidney outcomes and it would be important for these researchers to be aware of this interference in case serum creatinine is one of the outcomes of interest. In addition, NAC, by intravenous route, and at doses that would achieve high serum concentration (typically 150 mg/kg) are used in acetaminophen overdose and fulminant hepatitis.^8^ These patients are critically ill, and may develop AKI, with the assay interference potentially delaying the recognition of AKI. NAC has a relatively short half-life of 5.6 hours in adults and it is excreted renally.S1 The concentration of NAC needed to result in a significant interaction was very high at approximately 400 μg/ml NAC concentration, which would correspond to approximately 65 μmol/l. In a pharmacokinetic study, intravenous 25 mg/kg NAC (i.e., 6 times lower than the 150 mg/kg dose used in acetaminophen intoxication) resulted in a concentration of >180 μmol/l, suggesting that the interference we describe would be quite clinically relevant.

NAC has been used for prevention of AKI primarily in the contrast use setting, but also in the setting of postoperative AKI, and in chronic kidney disease. For CI-AKI, NAC use has fallen appropriately in disfavor once large trials reporting clinical outcomes did not report a benefit.^4^^,^^9^ A nephroprotective agent should prevent a rise in creatinine, but the initial small NAC trials reported a fall in creatinine, which does not make physiological sense. However, if NAC lowers serum creatinine by assay interference, as is demonstrated in the present study, this would clearly explain the conflicting results from the CI-AKI and NAC literature very well.

The other small body of literature in this area has tried to demonstrate the NAC effect by administering it to healthy adults, or those without any other exposure to AKI, and measuring creatinine before and after. In a systematic review, we demonstrated that this could be resolved, and the studies using the Jaffe method (−0.51 μmol/l, 95% confidence interval: −7.56 to 6.53) did not show a decrease in creatinine compared with the studies using the enzymatic assay (−3.24 μmol/l, 95% confidence interval: −6.29 to −0.28).S2 The present study extends these results by clearly demonstrating that the explanation lies in the concentration-dependent interference of NAC with the enzymatic assay. Indeed, from the previous systematic review, the greatest decrease in creatinine was reported with intravenous and high-dose NAC, which would achieve the high concentrations needed for significant artifactual reduction in creatinine. These findings are further supported by studies of interference mechanisms showing the Trinder reaction (bonding of 4-aminophenazone and a phenol derivative with H_2_O_2_ in the presence of peroxidase) is susceptible to inhibition by NAC in dose-dependent fashion.S3

Based on the results of this study, very high concentrations (>400 μg/ml) of NAC result in a significant negative bias (>10%) for enzymatic method for measurement of creatinine. There is no interference seen with the Jaffe method, nor with other kidney function measures such as cystatin-C and beta-trace protein. This information provides a satisfactory closure to the hitherto unexplained heterogeneity in the NAC and CI-AKI literature, and is also an important aspect to consider for ongoing NAC research and use in other settings.

## Disclosure

All the authors declared no competing interests.

## Supplementary Material

**Supplementary Methods**

**Figure S1.** Effects of NAC on Jaffe method.

**Figure S2.** Effects of NAC on different beta-trace proteins.

**Figure S3.** Effects of NAC on Cystatin-C.

Supplementary References
