# Supplementary material for: N-Acetylcysteine Interference With Creatinine Measurement: An In Vitro Analysis
Source: Kidney Int Rep. 2021 Apr 19;6(7):1973–6. doi: 10.1016/j.ekir.2021.04.006 (PMC8258456; doi:10.1016/j.ekir.2021.04.006)
Supplement: Supplementary File (PDF) [file mmc1.pdf]

Supplementary Methods

Objective

The objective of this study was to determine the effect of varying concentrations of NAC on measurement of kidney function: creatinine by enzymatic and Jaffe method, cystatin-C, and beta-trace protein by adding measured quantities of NAC to blood samples directly (using discard samples).

Study design and setting

The study design is a single centre, *in vitro* study conducted in a large tertiary care hospital in Canada.

Study population

There was no patient contact for this study since the testing used waste blood specimens. Patients' health records were accessed to identify CKD stages. Age, gender and creatinine level are required to calculate estimated glomerular filtration rate (eGFR) which is necessary for CKD staging.

To address the effects from a diversity of patients, blood samples included those from patients with low (50 µmol/L) slightly elevated (100 µmol/L) and very high (200 µmol/L) creatinine concentrations aiming for 3-5 replicates for each patient type for a total of 20-30 individual waste blood samples. After the samples were selected, a pool of each was created and the samples were de-identified. A 500 uL volume was needed for each sample (including dead volume for each samples for both Vista and VITROS® (Ortho Clinical Diagnostics); and dead volume for BN^{TM}II System (Siemens Healthineers).

Measurement

Waste blood specimens (plasma, collected in BD Vacutainer- PST plasma separation tubes and separated according to the manufacturer's instructions) were used to test the effect of NAC on various biochemical tests including: creatinine, cystatin-C, and beta-trace protein. Low, medium, and high creatinine sample pools with known concentration of analytes of interests were prepared from waste blood specimens (plasma). The three

pools were spiked with varying concentrations (0-2000 µg/mL) of NAC (20% solution of Acetylcysteine; Hospira Inc., Lake Forest. Il). Biochemical tests routinely used to identify kidney function were measured including: creatinine (enzymatic and non-enzymatic) with approximately 50, 100, 200 µmol/L, cystatin-C with 1, 2, 5 mg/L, and beta-trace protein with 1, 2, 5 mg/L.

Analytic plan

The results were analyzed by examining the effect of NAC concentration on the recovery and quantitative serum creatinine measurements relative to baseline. Interference plots were created to visualize and was characterized with respect to extent and effect. Results were illustrated in graphs as recovery plots and basic statistics used to assess the significance of observed effects. A 10% bias was pre-defined as clinically significance interference. All analysis was done with the statistical programming language R Core Team software (version 3.6.3, R Foundation for Statistical Computing, Vienna, Austria). Briefly, linear regression was done for each concentration versus recovery and visually inspected for fit. The threshold for 10% bias was established by interpolation between points using the fit describe above.

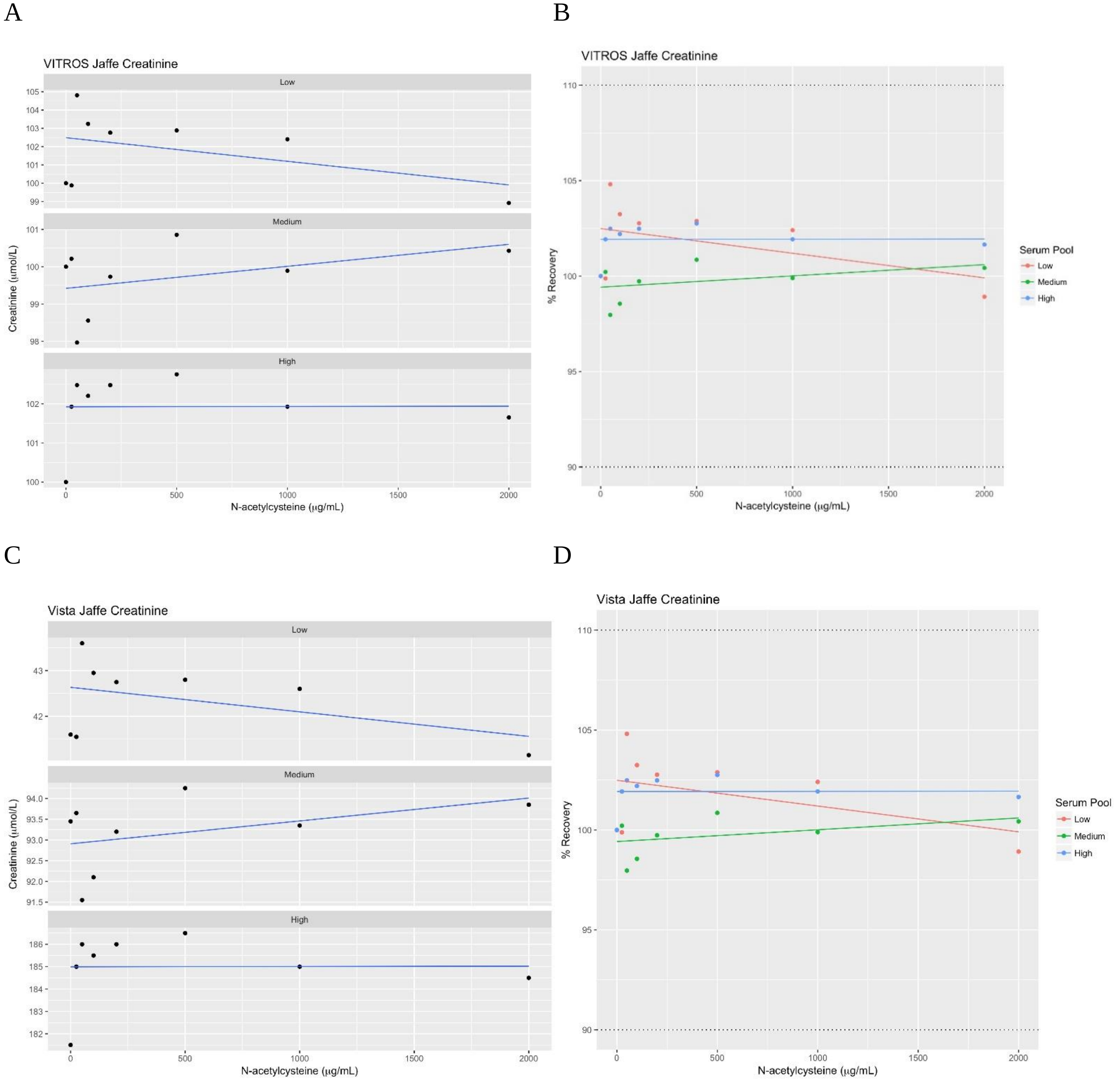

Figure S1. Effect of NAC on Jaffe method.

A. Effect of N-acetylcysteine on the absolute concentration of the Ortho Clinical Diagnostic Jaffe creatinine method.

B. Recovery of Jaffe creatinine method with NAC.

C. Effect of N-acetylcysteine on the absolute concentration of the Siemens Jaffe creatinine method.

D. Recovery of Siemens Jaffe creatinine methods with NAC.

A B

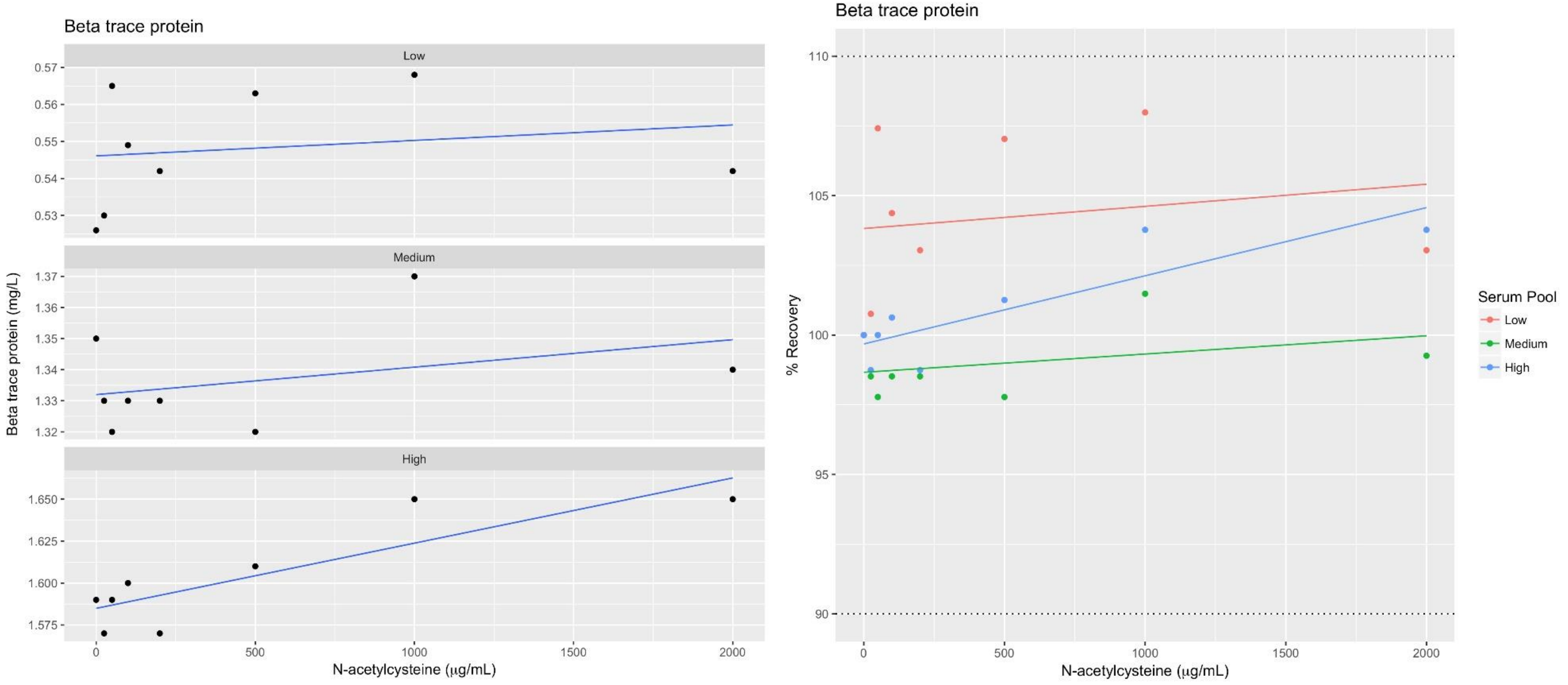

Figure S2. Effect of NAC on different beta-trace protein.

A. Effect of N-acetylcysteine on the absolute beta-trace protein concentration.

B. Recovery of beta-trace protein with NAC. Beta-trace protein was analyzed on the Siemens BNII Prospect analyzer according to the manufacture's instructions.

A B

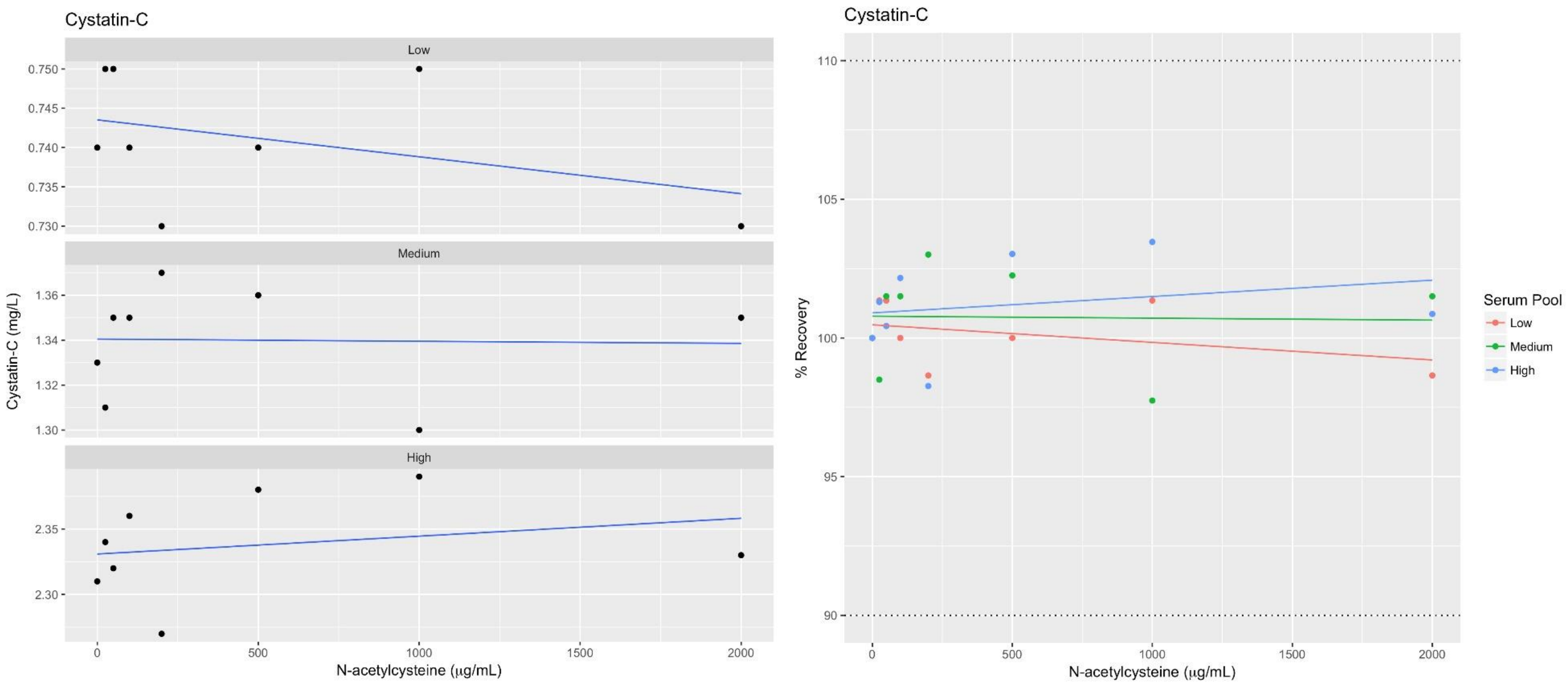

Figure S3. Effect of NAC on Cystatin-C.

A. Effect of N-acetylcysteine on the absolute cystatin-C concentration.

B. Recovery of cystatin-C with addition of NAC.

Supplementary References

S1 Sansone RA, Sansone LA. Getting a Knack for NAC: N-Acetyl-Cysteine. *Innov Clin Neurosci.* 2011;8(1):10-14.

S2 Huang JW, Lahey B, Clarkin OJ, et al. A Systematic Review of the Effect of N-Acetylcysteine on Serum Creatinine and Cystatin C Measurements. *Kidney International Reports.* 2020.

S3 Genzen JR, Hunsaker JJ, Nelson LS, Faine BA, Krasowski MD. N-acetylcysteine interference of Trinder-based assays. *Clinical Biochemistry.* 2016;49(1):100-104.
